# Price-based regulation of oligopolistic markets under discrete choice models of demand

**DOI:** 10.1007/s11116-021-10217-0

**Published:** 2021-08-09

**Authors:** Stefano Bortolomiol, Virginie Lurkin, Michel Bierlaire

**Affiliations:** 1 School of Architecture, Civil and Environmental Engineering, École Polytechnique Fédérale de Lausanne, Lausanne, Switzerland; 2 Department of Industrial Engineering and Innovation Science, Eindhoven University of Technology, Eindhoven, The Netherlands; 3 Faculty of Business and Economics, University of Lausanne, Lausanne, Switzerland

**Keywords:** Equilibrium, Regulation, Discrete choice modeling

## Abstract

We propose a framework to find optimal price-based policies to regulate markets characterized by oligopolistic competition and in which consumers make a discrete choice among a finite set of alternatives. The framework accommodates general discrete choice models available in the literature in order to capture heterogeneous consumer behavior. In our work, consumers are utility maximizers and are modeled according to random utility theory. Suppliers are modeled as profit maximizers, according to the traditional microeconomic treatment. Market competition is modeled as a non-cooperative game, for which an approximate equilibrium solution is sought. Finally, the regulator can affect the behavior of all other agents by giving subsidies or imposing taxes to consumers. In transport markets, economic instruments might target specific alternatives, to reduce externalities such as congestion or emissions, or specific segments of the population, to achieve social welfare objectives. In public policy, different agents have different individual or social objectives, possibly conflicting, which must be taken into account within a social welfare function. We present a mixed integer optimization model to find optimal policies subject to supplier profit maximization and consumer utility maximization constraints. Then, we propose a model-based heuristic approach based on the fixed-point iteration algorithm that finds an approximate equilibrium solution for the market. Numerical experiments on an intercity travel case study show how the regulator can optimize its decisions under different scenarios.

## Introduction

Public intervention in transport markets can be motivated by several phenomena. For decades it has been acknowledged that transportation is a source of negative externalities, two well-known cases of which are pollution and congestion. Policies to address these issues include road pricing (Button and Verhoef 1998; Anas and Lindsey 2011), taxes on fuel or on vehicle purchase (Fullerton and West 2002; Eliasson et al. 2018) and creation of low emission zones (De Borger and Proost 2013; Cullinane and Bergqvist 2014; Lurkin et al. 2021), among others. Currently, much attention is given to the contribution of the transport sector to the increase of greenhouse gas emissions which are a leading cause of climate change (IPCC 2014). Solutions that include a carbon tax are frequently proposed to reduce the negative impact of mobility on the environment. From a social perspective, a public entity might want to intervene in a transport market to incentivize mobility under certain circumstances. Indeed, improving mobility is often regarded as a means to increase economic output and enhance access to job opportunities or other activities (Van Goeverden et al. 2006; Guzman and Oviedo 2018). Additionally, many transport markets, alike other network industries such as energy and telecommunications, are natural monopolies where suppliers benefit from large economies of scale and consumers place greater value on large networks than on small ones (Farsi et al. 2007).

Public intervention can take many forms. In this work, we look at regulation. Regulation is defined as an indirect public intervention aimed at orienting actors towards some welfare goals (Ponti 2011). In this context, regulation can be seen as a middle way between a *command-and-control* approach and a pure *market competition* approach. Market regulation options are generally framed within competition and antitrust laws that exist at local, national and international level and determine how a regulator can influence the market. One common approach to regulation is the use of economic instruments such as subsidies and taxes, which are the focus of this contribution.

It is well-known that the acceptability of economic instruments for public policy depends on the perceived fairness of the instruments and on their effects across the population (Maestre-Andrés et al. 2019). To this end, discrete choice theory constitutes a powerful framework to analyze demand at a disaggregate level by accounting for product differentiation and consumer behavioral heterogeneity (Ben-Akiva and Lerman 1985; Anderson et al. 1992). A large body of discrete choice modeling literature exists which describes complex disaggregate choice behavior. However, these models are generally aggregated before being included in models of competitive markets (McFadden and Reid 1975; Koppelman 1976; Berry et al. 1995). The reason is that only the simplest disaggregate demand models, which require several limiting assumptions, satisfy the equilibrium existence conditions. In all other general cases, equilibrium existence is not guaranteed, and no analytical approach can be used to find one (Morrow and Skerlos 2011; Aksoy-Pierson et al. 2013; Gallego and Wang 2014).

Pacheco Paneque et al. (2021) present a novel approach to integrate general discrete choice models in mixed integer linear optimization by relying on simulation to draw from the distribution of the error term of the utility function. This methodology is particularly suitable for choice models that do not have a closed-form expression of the choice probabilities, such as mixed logit and probit. Using this simulation-based technique, Bortolomiol et al. (2021) extend the analysis to competitive markets by introducing an algorithmic framework to find approximate equilibrium solutions of oligopolies in which demand is modeled at the disaggregate level. This generic framework accommodates observed heterogeneity (e.g. socio-economic characteristics) and unobserved heterogeneity (e.g. parameter distribution) at the demand level, multi-product offer by suppliers and price differentiation strategies.

In this paper, we build upon the contributions by Pacheco Paneque et al. (2021) and Bortolomiol et al. (2021) and we propose a framework to find optimal policies to regulate oligopolistic transport markets where demand is modeled at a disaggregate level using discrete choice models. In markets characterized by imperfect competition between suppliers and by heterogeneous consumer demand, regulation affects the strategic decisions of suppliers, which in turn are influenced by the preferences of the customers and by the decisions of their competitors. Our approach allows to exploit an estimated discrete choice model and include it by means of simulation in a model of regulated competition featuring heterogeneous demand, multi-product offer by suppliers and price differentiation. Only a few assumptions are made about the demand and the specification of the discrete choice model, in order to accommodate advanced choice models, such as mixtures of logit, multivariate extreme value models, multivariate probit models or hybrid choice models. The use of models that capture complex disaggregate choice behavior allows the regulator to account for product differentiation and consumer behavioral heterogeneity at the individual level, and therefore to better tailor its policies based on tradeoffs between different agents.

The remainder of this paper is organized as follows. Section “Literature review” positions our contribution with respect to the literature on social welfare in presence of discrete choice models of user behavior and of imperfect competition. Section “A framework for regulated competition with discrete choice models” presents our discrete choice-based optimization model for regulated competition. The model can be integrated in an algorithmic framework that finds approximate equilibrium solutions for the market. Section “Case study” illustrates numerical experiments performed on a case study representing an intercity travel market. Finally, section “Conclusion” summarizes the main findings and provides directions for future research.

## Literature review

Welfare economics is generally understood as the problem of achieving a social maximum derived from individual desires by comparing and ranking different social states (Arrow 1951). If we postulate that interpersonal comparisons of utilities are meaningful, then value judgements are required to define a relation between utilities of different individuals and to aggregate them into a mathematical formula measuring social welfare. The necessity and the appropriateness of comparing gains of certain individuals with losses of other individuals when evaluating economic policies are central in the seminal works by Pareto (1906), Bergson (1938) and Samuelson (1948). Later studies look at the interdependencies between an individual’s social welfare function, representing ethical preferences, and their own utility function, representing personal tastes (Harsanyi 1955; Sen 1977), and formalize interpersonal comparability through social welfare functionals (d’Aspremont and Gevers 2002; Sen 2017). Social welfare functionals are flexible enough to incorporate many approaches to public policy, allowing not only descriptive but also normative analyses. Indeed, the study of social welfare has expanded to include subjective well-being, distributional preferences and intergenerational equity (Fleurbaey 2009).

Welfare economics uses social welfare functions to aggregate individual consumer behavior, which is generally modeled as the choice of a bundle of continuous goods subject to a budget constraint. Complementing the continuous case, the theory of discrete choice modeling considers behavioral situations in which an agent makes a choice from a finite set of discrete alternatives (Ben-Akiva and Lerman 1985). Discrete choice models account for consumer behavioral heterogeneity at the disaggregate level. As such, these models allow for complex and precise representations of individual behavior by means of utility functions that capture tastes and socio-economic characteristics.

Small and Rosen (1981) discuss how conventional methods of applied welfare economics can be generalized to handle random utility models of discrete choices. The authors recognize that welfare judgments are of paramount interest when analyzing taxes and subsidies in some markets for which discrete choice models are used. They conclude that welfare effects can be derived directly from micro data using the utility functions, avoiding the explicit use of aggregate demand functions, which are not normally obtained in closed form. In particular, consumer surplus can be expressed in different forms depending on the random term distributional assumptions. One of these forms is the log sum metric, utilized in the case of multivariate extreme value distribution. Batley and Ibáñez (2013) analyze the assumptions underlying the approach by Small and Rosen (1981). In particular, they show that the consumer surplus measure requires income effects of price and income changes to be equal to zero, thus excluding the possibility to have heterogeneous marginal utilities of income, which causes path dependence. Notwithstanding this limitation, the literature on welfare measurements using discrete choice models has largely followed the Marshallian framework (Hess et al. 2018). Alternative approaches that account for non-constant marginal utility of income rely on the Hicksian compensating variation (Hau 1985; Jara-Díaz and Videla 1990; McFadden 1995; Morey et al. 2003; Batley and Dekker 2019). However, both analytical and simulation-based methods come with a substantial computational burden, and this is the main reason for their limited use in practical applications to date. Finally, another method to account for different marginal utilities of income is proposed by Hau (1986), who modifies the approach by Small and Rosen (1981) to incorporate explicit value judgements by assigning distributional weights to segments of the population. The methodology is then used to carry out a transport infrastructure cost-benefit analysis where the population is stratified by income.

In prescriptive studies, we encounter some works whose goal is to design optimal fares, taxes or subsidies using a model of discrete choice. De Borger (2000) presents a model to determine a welfare-optimal two-part tariff under logit model of discrete choice, subject to budgetary constraints and distributional preferences. The fixed and the variable components of the tariff mimic the choices of ownership of a vehicle and quantity of consumption in terms of traveled kilometers. The expected value of the maximum utility for the logit model is obtained using the log sum welfare measure, interpreted as consumer surplus up to a constant, as in Small and Rosen (1981). It is shown that this methodology can be generalized to other classes of discrete choice models, for which there is no closed-form solution for the objective function, but no information is provided about computational tractability. A similar approach is followed in De Borger and Mayeres (2007), where nested constant elasticity of substitution utility functions are used. Borndörfer et al. (2012) propose a non-linear formulation to optimize fares on a public transport network. The demand functions take into account spatial heterogeneity in terms of origin-destination pairs and are based on a logit model to compromise between model accuracy and computability. Various objective functions are proposed to allow for the maximization of revenue, profit, demand, user benefit and social welfare.

The assumption that consumers are the only agents who react to welfare-maximizing policies is limiting when studying a regulated oligopolistic market where suppliers have market power and have objectives that conflict with those of the regulator. Indeed, in the latter case the outcome is determined jointly by all decision-makers, and the strategic behavior of firms in oligopolies is usually modeled using game theory (Fudenberg and Tirole 1991; Osborne and Rubinstein 1994). In a non-cooperative game, a Nash equilibrium solution is defined as a state in which no player can improve its payoff by unilaterally changing their decision (Nash 1951). Mathematically, an equilibrium is guaranteed to exist only if a number of conditions related to continuity, differentiability and convexity are satisfied for the demand, cost and profit functions (Murphy et al. 1982). In particular, these requirements pose limitations on the demand function. For this reason, the works that include discrete choice models in models of competitive markets and welfare analysis rely on simplifying assumptions that pose limitations on the model specification. Two examples are provided. Anderson et al. (2001) propose a welfare analysis of the efficiency of ad valorem and unit taxes in an imperfectly competitive market where each firm produces a homogeneous good. Within their analysis of Bertrand competition, the authors use a logit model to capture unobserved heterogeneity across the population and suggest that it is possible to extend the results to the case of differentiated products and to multiproduct firms. Ivaldi and Vibes (2008) analyze intermodal and intramodal price competition in a single origin-destination intercity passenger transport market where each firm controls one product, products are differentiated and unobserved demand heterogeneity is captured by a nested logit model. In this context, a unique Nash equilibrium solution exists and can be found by solving the first-order conditions of the firms’ profit maximization problem. The welfare effects of a kerosene tax are also evaluated by means of a sensitivity analysis.

These limitations on the demand function pose a severe restriction on the discrete choice model specification, and therefore on the possibility to conduct a truly disaggregate analysis and to develop disaggregate policies. We take a complementary stance and present a methodology that is applicable to complex discrete choice models at the expense of pure equilibrium existence conditions. With our work, we leverage on simulation to propose a comprehensive model that integrates general discrete choice models into a game-theoretic model of regulated competition, where the regulator aims to optimize a cardinal social welfare function which admits interpersonal comparisons of utility. In this context, the added value of our framework is twofold. First, it allows to generate a more precise representation of demand by modeling both observed and unobserved heterogeneity at a disaggregate level. Secondly, it allows to develop policies that leverage on disaggregate demand models to target specific segments of the population.

## A framework for regulated competition with discrete choice models

We consider a regulated competitive market where a number of different products are offered to a population by two or more suppliers that have market power.

On the demand side, let *N* represent the set of heterogeneous consumers (or groups of consumers), who are assumed to be utility maximizers, and let *I* indicate the discrete and finite set of alternatives available in the market. Utility functions $U_{in}$ are defined for each consumer or group of homogeneous consumers $n \in N$ and alternative $i \in I$. According to random utility theory (Manski 1977), $U_{in}$ can be decomposed into a systematic component $V_{in}$ which includes all that is observed by the analyst, accounting for the socio-economic characteristics and tastes of the individual and for the attributes of the alternative, and a random term $\varepsilon _{in}$ which captures the uncertainties caused by unobserved attributes and unobserved taste variations.

The deterministic part *V* can be generically expressed as

1 $$\begin{aligned} V_{in} = \beta _{p_{in}} p_{in} + q_{in}, \end{aligned}$$

where $p_{in}$ is the price paid by consumer *n* when purchasing alternative *i*, $\beta _{p_{in}}$ is a pre-estimated parameter of the discrete choice model and $q_{in}$ is the scalar product of the vectors of all the exogenous variables of the choice model and the corresponding pre-estimated parameters.

On the supply side, let *K* represent the set of suppliers and let $I_k \subset I$ indicate the subset of alternatives controlled by each supplier $k \in K$. We impose that $\cup _{k \in K} I_k \subset I$, in order to allow customers to leave the market without purchasing. We assume that each supplier solves a choice-based optimization problem, modeled in the form of a mixed integer optimization problem, aimed at finding the strategy that maximizes its profits. We define as ${\mathcal {S}}_k$ the set of strategies that can be selected by supplier *k*. A strategy consists in a vector (or bundle) $p^S$ of decisions about all prices $p_{in}^S$, potentially differentiated for each (class of) consumer $n \in N$ and alternative $i \in I_k$.

Furthermore, we assume the existence of a regulator, which is an entity that can use economic instruments, that is, subsidies and taxes, to influence the behavior of the other agents, thus modifying the equilibrium outcome of the market. In a general case, we assume that the regulator has a budget *B* which is available to finance some policies. The policies set by the regulator affect the price $p_{in}$ paid by consumer (group) *n* for alternative *i*, which in return affects the utilities $U_{in}$ of the consumers.

By combining the decisions of the regulator and of the suppliers, we obtain the prices $p_{in}$ paid by the consumers, which can be decomposed as

2 $$\begin{aligned} p_{in} = p_{in}^S + t_{in}, \end{aligned}$$

where $p_{in}^S$ is the revenue made by the supplier in case of purchase and $t_{in}$ is the tax or the subsidy set by the regulator. If $t_{in} > 0$, then a tax is imposed on the purchase of alternative *i* by customer *n*. If $t_{in} < 0$, then a subsidy is offered for the same purchase.

Then, we can write an optimization problem from the point of view of the regulator, whose goal is to maximize a social welfare function (SWF) at equilibrium. The decision variables of the regulator problem are the continuous $t_{in}$ variables, potentially differentiated for each (class of) consumer $n \in N$ and alternative $i \in I_k$. For modeling purposes, we define bounds on the tax and subsidy levels, expressed by the non-negative parameters $M_{in}^t$ and $M_{in}^s$, such that $-M_{in}^s \le t_{in} \le M_{in}^t$.

Let us now look in detail at the different components of the modeling framework.

### Constraints

Three sets of conditions need to be enforced to model the common behavioral assumptions about consumers and suppliers: (i) utility maximization conditions; (ii) profit maximization conditions; (iii) equilibrium conditions. On top of them, other problem-specific constraints can be defined to model specific market features.

#### Utility maximization

Concerning utility maximization, we impose a set of linear constraints by applying the simulation-based linearization approach proposed by Pacheco Paneque et al. (2021). A set *R* of independent draws are extracted from the known error term distribution of the discrete choice model for each $n \in N$ and $i \in I$, corresponding to different behavioral scenarios. For each scenario $r \in R$, the drawn error term parameter $\xi _{inr}$ is included in the utility function as follows:

3 $$\begin{aligned} U_{inr} = V_{in} + \xi _{inr}, \end{aligned}$$

and consumers deterministically choose the alternative with the highest utility. This means that the utility of the chosen alternative is equal to

4 $$\begin{aligned} U_{nr}^{{\textit{max}}} = \max _{j \in I} U_{jnr}. \end{aligned}$$

Then, we can express the deterministic choice of consumer $n \in N$ in a specific scenario $r \in R$ using the binary variable $P_{inr}$ as follows:

5 $$\begin{aligned} P_{inr} = {\left\{ \begin{array}{ll} 1 \quad \text {if}\; U_{inr} = U_{nr}^{{\textit{max}}},\\ 0 \quad \text {otherwise.} \end{array}\right. } \end{aligned}$$

For each consumer *n* and scenario *r*, expressions (3–5) can be reformulated as a set of linear constraints in the following manner:

6 $$\begin{aligned}&U_{inr} = \beta _{p_{in}} (p_{in}^S + t_{in}) + q_{in} + \xi _{inr}&\forall i \in I, \end{aligned}$$

7 $$\begin{aligned}&U_{inr} \le U_{nr}^{{\textit{max}}}&\forall i \in I, \end{aligned}$$

8 $$\begin{aligned}&U_{nr}^{{\textit{max}}} \le U_{inr} + M_{U} (1 - P_{inr})&\forall i \in I, \end{aligned}$$

9 $$\begin{aligned}&\sum _{i \in I} P_{inr} = 1&\forall i \in I, \end{aligned}$$

10 $$\begin{aligned}&P_{inr} \in \{0,1\}&\forall i \in I. \end{aligned}$$

Constraints (6) define the utility functions. Constraints (7–9) state that the consumer chooses the alternative with the highest utility. The parameter $M_U$ must be sufficiently big to inactivate constraints (8) for all alternatives that are not chosen. A valid value of $M_U$ is the difference between the highest and the lowest utility across all alternatives, which itself depends on the bounds on prices, taxes and subsidies. Constraints (10) are the domain constraints.

Over a sufficiently large number of draws, we obtain the choice probabilities

11 $$\begin{aligned} P_{in} = \frac{\sum _{r \in R} P_{inr}}{\mid R \mid } \end{aligned}$$

and the expected maximum utilities

12 $$\begin{aligned} {\textit{EMU}}_{n} = \frac{\sum _{r \in R} U_{nr}^{{\textit{max}}}}{\mid R \mid }. \end{aligned}$$

Numerical experiments by Pacheco Paneque et al. (2021) show that good approximations of the choice probabilities and of the expected maximum utility can be obtained with a fairly low number of draws.

#### Profit maximization

The profit maximization problem of each supplier can be expressed through the following mixed integer linear optimization model:

13 $$\begin{aligned} \max _{p_k^S} \quad \pi _{k} = \sum _{i \in I_k} \sum _{n \in N} \theta _n P_{in} p_{in}^S, \end{aligned}$$

14 $$\begin{aligned} {\textit{s.t.}} \quad \text {constraints (6--10)}. \end{aligned}$$

In (13), $p_{in}^S$ is the revenue obtained from the sale of product *i* to consumer *n* [see Eq. (2)], $P_{in}$ is the probability that consumer group *n* chooses alternative $i \in I_k$ [see Eq. (11)], and $\theta _n$ is the size of group *n*, i.e. the number of individuals with homogeneous socioeconomic characteristics. The supplier optimization problem constitutes lower-level optimization constraints for the regulator optimization problem.

#### Equilibrium conditions

Consistently with Bortolomiol et al. (2021), we use $\varepsilon$-equilibrium conditions to identify stationary states of the system in which no competitor can increase its profit to more than $1 + \varepsilon$ times its current payoff by unilaterally changing its strategy. Formally, let us consider a market state $S = (p^S,t)$, which is defined by the strategies $p_k^S$ of all suppliers $k \in K$ and the vector of all taxes (or subsidies) $t_{in}$ set by the regulator. Let us define as $\pi _{k}(S)$ the expected profit of supplier *k* in state *S* and as $\pi _{k}^{{\textit{max}}}(S_{-k})$ the expected profit obtained by supplier *k* when best responding to state $S_{-k}$, defined by the strategies of the regulator and all suppliers except *k*. Then, *S* is an $\varepsilon$-equilibrium if

15 $$\begin{aligned} \pi _{k}^{{\textit{max}}}(S_{-k}) \le (1 + \varepsilon ) \pi _{k}(S) \quad \forall k \in K. \end{aligned}$$

#### Problem-specific constraints

Other problem-specific constraints can be integrated in a mixed integer linear formulation. For instance, many competition laws impose that the taxation or subsidization of products sold by competing suppliers must be fair, meaning that no competitive advantage must arise due to the intervention of the government in the market. On the supplier side, constraints can be included to ensure that demand for an alternative does not exceed capacity. This can be achieved by means of exogenous priority rules that simulate the arrival process of customers, as shown in Binder et al. (2017). With this technique, it is also possible to model price differentiation strategies based on the time of booking. On the consumer side, price bounds can be set to define limits for price discrimination across different population groups. All these sets of constraints reduce the feasible set of solutions in the optimization problems of the regulator and of the suppliers. Their effect in terms of computational time and resulting equilibrium is generally problem-dependent. In the case study presented in section “Case study”, we show how our modeling framework can integrate some of these constraints. Other constraints, including capacity constraints and the related congestion effects, come with additional integrality constraints or non-linearities, which require the design of ad-hoc algorithms to be tackled (Pacheco Paneque 2020).

### Objective function

The objective of the market regulator is to maximize a cardinal social welfare function (SWF). We assume that this function takes into account the following components: (i) expected maximum utilities of the consumers; (ii) expected profits of the suppliers; (iii) market externalities; (iv) cost of policy implementation. To allow for comparability of different terms, we choose to monetize all values that are measured in non-monetary terms.

*Expected utilities* As shown in (12), expected maximum utilities can be derived directly from the discrete choice-based optimization model. The utility functions must be converted from preference space into the equivalent formulation in willingness-to-pay space, as discussed by Train and Weeks (2005). We consider linear income effects, thus adhering to the assumption outlined by Batley and Ibáñez (2013) under which the framework by Small and Rosen (1981) is consistent with economic theory. This means that consumer surplus can be obtained by dividing the expected maximum utility by a cost coefficient $\beta _{p}$, which corresponds to the constant marginal utility of income.

This component of the SWF can then be written as follows:

16 $$\begin{aligned} {\textit{SWF}}_U = \sum _{n \in N} \theta _n \frac{{\textit{EMU}}_{n}}{\beta _{p}}. \end{aligned}$$

*Expected profits* The sum of the expected profits of the suppliers can also be obtained directly from the discrete choice-based optimization model. The expected profits of supplier *k* are defined in Eq. (13), so the sum of the expected profits is simply

17 $$\begin{aligned} {\textit{SWF}}_{\pi } = \sum _{k \in K} \pi _{k}. \end{aligned}$$

*Externalities* In transportation, externalities can be generally expressed as a function of demand, which is itself derived from the choice probabilities, as follows:

18 $$\begin{aligned} d_i = \sum _{n \in N} \theta _n P_{in}. \end{aligned}$$

In the case of environmental externalities, we may approximate ${\mathrm {CO}}_{2}$ and other emissions to a linear function of demand:

19 $$\begin{aligned} {\textit{SWF}}_{E} = - \sum _{i \in I} c_i d_i, \end{aligned}$$

where $d_i$ is the demand for alternative *i* and $c_i$ is a parameter representing the monetized cost of emissions per person choosing alternative *i*, which can be expressed as

20 $$\begin{aligned} c_i = \ell _{i} \cdot e_i \cdot {\textit{SCC}}, \end{aligned}$$

where $\ell _{i}$ is the distance traveled if alternative *i* is chosen [km], $e_i$ is the ${\mathrm {CO}}_{2}$ emissions produced per unit of distance when traveling with alternative *i* [ton/km] and ${\textit{SCC}}$ is the social cost per unit of carbon emission [monetary unit/ton].

In the case of negative externalities caused by road congestion, it is well-known that a non-linear relation exists between traffic volume and total travel time, which also affects the utility of the users. This requires a fixed-point iteration approach to be used in order to reach lower-level user equilibrium.

*Public budget* The monetary impact of the policy for the regulator is given by the difference between the taxes that are collected and the subsidies that are handed out from and to consumers, and is therefore conditional on their choices. The budget can be expressed as the sum of the products of the continuous tax variables $t_{in}$ and the binary choice variables $P_{inr}$:

21 $$\begin{aligned} {\textit{SWF}}_{B} = \sum _{i \in I} \sum _{n \in N} \theta _n \frac{\sum _{r \in R} P_{inr}}{\mid R \mid } t_{in}. \end{aligned}$$

Expressions (16), (17), (19) and (21) capture different aspects of welfare which are relevant in policy-making. They can be integrated in a unique objective function, or alternatively they can be treated as different objectives in a multi-objective optimization problem. In the rest of this work, we follow the former approach.

### The full model

By combining the constraints presented in section “Constraints” and the objective function presented in section “Objective function”, we obtain the following mixed integer optimization model that maximizes the SWF as a function of the taxation and subsidization chosen by the regulator:

22 $$\begin{aligned} \max _{t} \quad {\textit{SWF}}(t,p^S) \end{aligned}$$

23 $$\begin{aligned} {\textit{s.t.}} \quad -M_{in}^s \le t_{in} \le M_{in}^t \quad \forall i \in I, \forall n \in N, \end{aligned}$$

24 $$\begin{aligned} t'_{in} = t_{in} + M_{in}^s \quad \forall i \in I, \forall n \in N, \end{aligned}$$

25 $$\begin{aligned} 0 \le \gamma '_{inr} \le (M_{in}^s + M_{in}^t) P_{inr} \quad \forall i \in I, \forall n \in N, \forall r \in R, \end{aligned}$$

26 $$\begin{aligned} t'_{in} - (M_{in}^s + M_{in}^t) (1 - P_{inr}) \le \gamma '_{inr} \le t'_{in} \quad \forall i \in I, \forall n \in N, \forall r \in R, \end{aligned}$$

27 $$\begin{aligned} -M_{in}^s \le \gamma _{inr} \le M_{in}^t P_{inr} \quad \forall i \in I, \forall n \in N, \forall r \in R, \end{aligned}$$

28 $$\begin{aligned} \gamma '_{inr} - M_{in}^s - (M_{in}^s + M_{in}^t) (1 - P_{inr}) \le \gamma _{inr} \le \gamma '_{inr} - M_{in}^s \quad \forall i \in I, \forall n \in N, \forall r \in R, \end{aligned}$$

29 $$\begin{aligned} \sum _{i \in I} \sum _{n \in N} \sum _{r \in R} \gamma _{inr} \le B \end{aligned}$$

30 $$\begin{aligned} \pi _{k} = \max _{p_k^S} \sum _{i \in I_k} \sum _{n \in N} \theta _n P_{in} p_{in}^{s} \quad \forall k \in K, \end{aligned}$$

31 $$\begin{aligned} {\textit{s.t.}} \quad U_{inr} = \beta _{p_{in}} (p_{in}^S + t_{in}) + q_{in} + \xi _{inr} \quad \forall i \in I, \forall n \in N, \forall r \in R, \end{aligned}$$

32 $$\begin{aligned} U_{inr} \le {\textit{EMU}}_{nr} \quad \forall i \in I, \forall n \in N, \forall r \in R, \end{aligned}$$

33 $$\begin{aligned} {\textit{EMU}}_{nr} \le U_{inr} + M_{U} (1 - P_{inr}) \quad \forall i \in I, \forall n \in N, \forall r \in R, \end{aligned}$$

34 $$\begin{aligned} \sum _{i \in I} P_{inr} = 1 \quad \forall i \in I, \forall n \in N, \forall r \in R, \end{aligned}$$

35 $$\begin{aligned} P_{inr} \in \{0,1\} \quad \forall i \in I, \forall n \in N, \forall r \in R. \end{aligned}$$

The objective function (22) maximizes the social welfare function defined by the regulator. Constraints (23) impose that the subsidies and taxes set by the regulator respect the given bounds. Constraints (24) define the non-negative variables $t'_{in}$ by means of a transformation. Notice that the parameters $M_{in}^s$ and $M_{in}^t$, which are upper bounds representing the maximum possible values for the subsidies and taxes, are necessary to linearize the product of the binary choice variable $P_{inr}$ and the continuous variable $t_{in}$, which is done in constraints (25–28). More specifically, constraints (25–26) say that each auxiliary variable $\gamma '_{inr}$ is equal to $t'_{in}$ if $P_{inr}$ is equal to 1 and is equal to 0 if $P_{inr}$ is equal to 0, while constraints (27–28) say that $\gamma _{inr}$ is equal to the product $P_{inr} \cdot t_{in}$. Constraint (29) ensures that the budget of the regulator is respected. The expressions (30) represent the objective functions of the lower-level problem, enforcing the profit maximization condition on all suppliers. Finally, constraints (31–35) are the utility maximization constraints (6–10).

### Model-based heuristic framework

To solve (22–35), we propose a model-based heuristic approach based on the fixed-point iteration algorithm which finds optimal policies subject to approximate equilibrium conditions. More specifically, two mixed integer linear optimization models are derived from (22–35): the supplier’s profit maximization model (30–35), where the regulator’s decisions as well as the prices of all other suppliers are fixed, and a modified regulator’s welfare maximization model (22–29) + (31–35) where all supply prices are fixed and the optimization constraints (30) are not enforced. Algorithm 1 presents the pseudocode of the proposed solution.

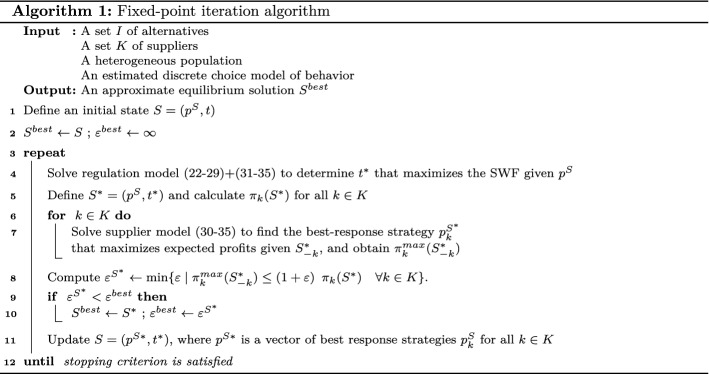



Starting from an initial state $S = (p^S,t)$, the fixed-point iteration algorithm works as follows. First, the regulator solves model (22–29) + (31–35) to find $t^*$ that maximizes the SWF given $p^S$. A new state $S^* = (p^S,t^*)$ is obtained, for which the expected profits $\pi _{k}(S^*)$ are computed. Then, each supplier solves model (30–35) given $t^*$ and $p_{-k}^{S^*}$ to obtain the best-response expected profits $\pi _{k}^{BR}(S^*)$. With all best-response profits, we can compute the value $\varepsilon ^{S^*}$ as follows:

$$\begin{aligned} \varepsilon ^{S^*} = \min \{\varepsilon \mid \pi _{k}^{{\textit{max}}}(S^*_{-k}) \le (1 + \varepsilon ) \pi _{k}(S^*) \quad \forall k \in K\}. \end{aligned}$$

This value gives information about the stability of an approximate equilibrium solution, that is, the higher $\varepsilon ^{S^*}$ is, the less stable the solution is, since there is a greater incentive for at least one supplier to move away from it. If $\varepsilon ^{S^*}$ is lower than the current best $\varepsilon$, this means that $S^*$ is the most stable solution found so far. Finally, state *S* is updated with the best-response strategies of all suppliers before restarting with a new iteration. When implementing the algorithm, checks can be made to track visited states and diversify the search of the solution space within the algorithm. The algorithm can be terminated based on different stopping criteria, such as finding an $\varepsilon$-equilibrium solution with $\varepsilon$ lower than a predefined target or reaching a maximum number of iterations.

Notice that, when using demand functions based on general disaggregate choice models, which are highly non-linear and non-convex, there is no pure equilibrium existence condition for the resulting problem, and no analytical method can be exploited to find a pure equilibrium solution. More generally, the existence of a $\varepsilon$-equilibrium solution cannot be proved for any given $\varepsilon$.

## Case study

In this section, we illustrate the model-based algorithmic framework presented in section “Model-based heuristic framework” on a case study for which a non-trivial discrete choice model of demand is taken from the literature (Cascetta and Coppola 2012).

### Data

We consider a competitive intercity travel market connecting two cities in a typical morning period. The travel distance between the two cities is assumed to be 1200 km for all travel modes. The market is served by an airline, a high-speed rail company and an intercity train company operating under public service obligations. Additionally, we include the possibility that customers use a private means of transport, which is modeled as an opt-out alternative. We endogenously model the pricing strategies of the airline and of the high-speed rail operator, which must decide on the prices at which to sell tickets for each scheduled departure time. The price of the intercity train and of private transport are assumed to be fixed and exogenously given. We also endogenously model the policies of the regulator, which decides on taxes or subsidies that lead to a welfare-maximizing outcome.

Table 1 shows the supply data used for the tests. Travelers can choose among six different alternative services to go from origin to destination within a given time period. The car alternative is modeled as exogenous option, i.e. all its attributes are assumed to be parameters of the problem, while train and air alternatives are modeled endogenously, that is, the two competing operators, each controlling two alternatives, and the regulator strategically determine prices and taxes or subsidies in response to the conditions of the market. The attributes that are included in the customer utility functions for the different alternatives are cost, in-vehicle travel time, waiting time, access time to and egress time from terminals, early or late arrival at destination with respect to the desired arrival time of the traveler.

**Table 1:** Attributes of all scheduled services for the analyzed problem instance

Alternative	0	1	2	3	4	5
Mode	Car	IC	Air	Air	HSR	HSR
Endogenous	No	No	Yes	Yes	Yes	Yes
Operator	–	–	2	2	1	1
Dep	–	23:00	7:30	9:30	4:30	8:30
Arr	–	9:00	9:00	11:00	10:30	14:30
TT	12 h	10 h	1 h 30’	1 h 30’	6 h	6 h
WT	–	–	1h	1h	–	–
Access	–	0–60′	30–60′	30–60′	0–60′	0–60′
Egress	–	0–30′	30-60′	30–60′	0–30′	0–30′
Price	120 €	60 €	$p_2$	$p_3$	$p_4$	$p_5$
Tax/subsidy	–	$t_{{\textit{TRAIN}}}$	$t_{{\textit{AIR}}}$	$t_{{\textit{AIR}}}$	$t_{{\textit{TRAIN}}}$	$t_{{\textit{TRAIN}}}$

Furthermore, we generate a synthetic population of 1000 travelers for the given OD pair. Individuals are characterized by a trip purpose (business or other), an income level (high or low), and a specific origin location (urban or rural) which leads to different access times to terminals. For the sake of the experiments, we assume that business travelers have a desired arrival time at destination between 9:00 and 12:00, which follows a non-uniform distribution: 50% of them desire to arrive between 9:00 and 10:00 (peak period), the rest between 10:00 and 12:00. Furthermore, we assume that all non-business travelers are indifferent to arrival time. The following demand patterns are to be mentioned: there is a higher proportion of high income and business travelers among urban travelers than among rural travelers; a part of business travelers are reimbursed and are therefore less price sensitive. We categorize the synthetic population in 12 groups of consumers, each having homogeneous socioeconomic characteristics. Table 2 represents the contingency table of the synthetic population.

**Table 2:** Contingency table for the synthetic population according to socio-economic characteristics

Group (*n*)	Size ($\theta _n$)	Trip purpose	Reimbursement	Income	Origin
1	350	Other	–	Low	Rural
2	332	Other	–	Low	Urban
3	37	Other	–	High	Rural
4	39	Other	–	High	Urban
5	9	Business	No	Low	Rural
6	24	Business	Yes	Low	Rural
7	16	Business	No	Low	Urban
8	68	Business	Yes	Low	Urban
9	5	Business	No	High	Rural
10	30	Business	Yes	High	Rural
11	21	Business	No	High	Urban
12	69	Business	Yes	High	Urban

The discrete choice model is derived from Cascetta and Coppola (2012), where a nested logit model is estimated from a RP/SP survey dataset collected in Italy at the national level. Table 3 illustrates the parameters used in our experiments. Two separate sets of parameters are considered for business trips and other trip purposes. Additionally, the cost parameters are mode-specific and interact with income, producing different values of travel time savings, which are reported in Table 4. Two nests $\mu _{{\textit{HSR}}}$ and $\mu _{{\textit{Air}}}$ capture the correlation between the scheduled services of the high-speed train operator and of the airline. Values without asterisk are taken as such from Cascetta and Coppola (2012). The $\beta _{{\textit{cost}}_{{\textit{car}}}}$ parameter for reimbursed business customers is derived by assuming that the ratio between the values of travel time of reimbursed and non-reimbursed business travelers by car is the same as by train. We have introduced an additional distinction between high income and low income travelers. This is done in order to test scenarios where public intervention is targeted to specific segments of the population. We have arbitrarily assumed that $\beta _{{\textit{cost}}}$ parameters of non-reimbursed business travelers and of other travelers from Cascetta and Coppola (2012) apply to our low income segment of the population. $\beta _{{\textit{cost}}}$ parameters for high income customers are derived by assuming that the ratio between the values of travel time of high income and low income customers is the same as in the SAMPERS long-distance model developed in Sweden and reported in Börjesson (2014).

We remark that the dataset used for the experiments and the derived results are hypothetical and do not represent real scenarios that are related to choices made by existing high-speed rail operators.

**Table 3:** Model coefficients derived from Cascetta and Coppola (2012)

$\beta$		Business travelers		Other purpose travelers	
$\mu _{{\textit{Air}}}$		1.086		1.106	
$\mu _{{\textit{HSR}}}$		1.190		1.333	
Travel time (min)		−0.0133		−0.0054	
Access/egress time (min)		−0.00555		−0.0103	
Early schedule delay (min)		−0.00188		−0.00677	
Late schedule delay (min)		−0.0130		−0.00617	

	Reimbursed	High income	Low income	High income	Low income
Cost car (euro)	−0.0222*	−0.0296*	−0.0527	−0.0228*	−0.0405
Cost Air (euro)	−0.0109	−0.0113*	−0.0201	−0.0109*	−0.0194
Cost IC (euro)	−0.0158	−0.0212*	−0.0377	−0.0097*	−0.0172
Cost HSR (euro)	−0.0120	−0.0160*	−0.0284	−0.0144*	−0.0256

**Table 4:** Values of travel time

Value of travel time	Reimbursed	High income	Low income	High income	Low income
Car (euro/h)	35.88*	26.95*	15.14	14.24*	8.00
Air (euro/h)	73.21	70.67*	39.70	29.73*	16.70
IC (euro/h)	50.51	37.68*	21.17	33.54*	18.84
HSR (euro/h)	66.50	50.02*	28.10	22.53*	12.66

### Numerical experiments

The framework described in section “A framework for regulated competition with discrete choice models” allows to model various policies and answer questions about the strategic behavior of all agents at equilibrium. Common regulatory policies include price-based instruments such as taxes and subsidies, but also other instruments such as emission allowances. With respect to demand, we can look at the effect of taxes and subsidies on the choices and the utilities for the population as a whole and for specific segments. With respect to supply, we can look at the changes in the pricing strategies and at the effect of regulation on profits. With respect to regulation and welfare, we can look at a social welfare function that combine consumers’ utilities, suppliers’ profits, public budget and environmental effects to understand tradeoffs between these different objectives.

*Base scenario* Initially, we assume that the regulator sets uniform taxes or subsidies across consumers. Likewise, the suppliers set uniform prices to all consumers, that is, there is no price differentiation. Two components of the SWF, which are the sum of the expected profits ${\textit{SWF}}_{\pi }$ and the public expenditure ${\textit{SWF}}_{B}$, are expressed in the monetary terms. The other two components, which are the sum of the expected utilities ${\textit{SWF}}_{U}$ and the externalities ${\textit{SWF}}_{E}$, must be converted into monetary units. To convert the expected utilities, we set the constant marginal utility of income to be the weighted average of the $\beta _{{\textit{cost}}}$ parameters across transport modes (see Table 3), with weights that reflect the market shares used in the phase of calibration. The resulting value is $-0.01832$. To monetize externalities, we use the social cost of carbon (SCC), defined as the monetary value of the damage caused by emitting one more unit of carbon at some point of time (Nordhaus 1994; Pearce 2003; Stern et al. 2006). The SCC is typically derived from integrated assessment models which require an assumption on the future path of ${\mathrm {CO}}_{2}$ concentration in the atmosphere. Although the range of SCC estimates available in the literature is quite broad, this indicator has been central in shaping climate policy and is extensively used within cost-benefit analyses. Here, we test values ranging between 100 and 300 € per ton of carbon.

Additionally, we define the upper bounds $M_{in}^s$ and $M_{in}^t$ to be equal to 30 € for all alternatives and customers, and we do not impose a public budget constraint.

Table 5 presents the approximate equilibrium solutions for five values of the SCC. These solutions are obtained by running Algorithm 1 with two possible stopping criteria: either a solution is found where no supplier can improve its profits by more than 1% by best-responding, i.e. $\varepsilon < 0.01$, or a maximum number of iterations (200 in our experiments) is reached. In the latter case, we report the solution with the lowest value of $\varepsilon$. Bortolomiol et al. (2021) provide a broader analysis of the approximate equilibrium solutions found by using a simulation-based framework. To obtain the choice probabilities through simulation, we use 200 draws from the error term distribution for each alternative *i* and customer *n*.

By looking at the strategic policy decisions of the regulator, we observe that air taxes and train subsidies are higher for higher values of the SCC parameter. For ${\textit{SCC}} \ge 250$ €/ton, the regulator sets the maximum level of intervention. This result reflects the relative importance of the cost of externalities with respect to all other objectives. On the suppliers’ side, we see that, for moderate public intervention, the fares set by the airline operator are higher than the fares offered by the high-speed rail operator. This choice is motivated by the travelers’ willingness to pay for large travel time savings. When air taxes and train subsidies increase, e.g. in the scenario with ${\textit{SCC}} = 200$ €/ton, the airline operator decreases its prices in order not to lose too many customers (i.e. high market share, lower markup). Contrarily, the optimal prices of the high-speed rail operator exhibit non-monotonic variations, for which different possible explanations coexist. In fact, it is important to notice that profit functions are generally multimodal in presence of disaggregate demand. On the one hand, similar profits can be obtained with different pricing strategies and market shares. On the other hand, when a supplier manages two or more alternatives, there is a form of internal competition between these alternatives, which is particularly relevant when the desired arrival time of the demand does not play a dominant role in the utility function, that is, they are nearly perfect substitutes. Notice that this type of analysis is very much instance-dependent and is influenced by factors such as similarities across alternatives, degree of heterogeneity across the population and size of the population groups, among others.

By analyzing the different components of the SWF, we observe that, when we increase the SCC, the increased relative weight of the emissions component causes a stronger public intervention. The negative sign of the ${\textit{SWF}}_{B}$ component indicates that the money distributed to subsidize rail alternatives is larger than the money collected from taxes on air tickets. We infer that this result is partially motivated by the fact that the utility specification of the used choice model includes mode-specific $\beta _{{\textit{cost}}}$ parameters. In this particular case, this means that a unitary price change on the train alternatives has a higher effect on the utility than a unitary price change on the air alternatives. In general, the appropriateness of using mode-specific cost parameters and the selection of a valid value for the marginal utility of income within our modeling framework are open questions for which further research should be conducted to derive more sound conclusions.

Table 6 shows the impact of regulation on utilities and market shares at a disaggregate level, according to the defined income-segments. The comparison between the scenarios with ${\textit{SCC}} = 100$ €/ton and ${\textit{SCC}} = 300$ €/ton shows that heavier public intervention causes the highest air-to-rail modal shift to occur among low income people, who are more price sensitive. Furthermore, the prevalence of subsidies over taxes in these scenarios has a positive effect on the segmented ${\textit{SWF}}_{U}$ of both high income and low income travelers. The values of ${\textit{SWF}}_{U}$ for ${\textit{SCC}} = 100$ €/ton are set to 0 and used as a benchmark. Notice that all these analyses neglect all exogenous information such as the source of the public budget.

**Table 5:** Approximate equilibrium solutions for an objective function that maximizes the social welfare function with values of the SCC between 100 and 300 €/ton

SCC	$\varepsilon$	$t{\mathrm {CO}}_{2}$	Air prices		HSR prices		Regulation		Objective function			
			$r_2$	$r_3$	$r_4$	$r_5$	$t_{{\textit{TRAIN}}}$	$t_{{\textit{AIR}}}$	${\textit{SWF}}_{U}$	${\textit{SWF}}_{\pi }$	${\textit{SWF}}_{E}$	${\textit{SWF}}_{B}$
100	0.014	147.12	101.08	109.26	82.42	83.35	−14.61	2.26	0	90,598	−14,712	−6907
150	0.010	132.26	93.08	93.29	88.62	84.51	−29.68	12.73	8029	86,723	−19,838	−13,207
200	0.008	125.43	81.28	79.92	86.97	76.04	−29.90	26.80	10,516	79,984	−25,086	−9588
250	0.011	123.62	80.54	79.53	86.47	79.44	−30.00	30.00	9016	80,315	−30,906	−8955
300	0.010	118.39	79.96	90.34	86.20	76.49	−30.00	30.00	8804	80,813	−35,517	−10,167

**Table 6:** Segmented monetized utilities and market shares for high and low income customers with values of the SCC between 100 and 300 €/ton

SCC	Monetized utilities		Market shares high income						Market shares low income					
	${\textit{SWF}}_{U}^H$	${\textit{SWF}}_{U}^L$	Car	IC	Air1	Air2	HSR1	HSR2	Car	IC	Air1	Air2	HSR1	HSR2
100	0	0	0.053	0.042	0.329	0.159	0.345	0.073	0.024	0.125	0.245	0.168	0.261	0.177
150	717	7312	0.046	0.046	0.301	0.162	0.366	0.079	0.015	0.141	0.198	0.153	0.275	0.218
200	832	9684	0.046	0.045	0.297	0.157	0.365	0.090	0.015	0.132	0.176	0.145	0.277	0.255
250	684	8332	0.047	0.045	0.290	0.154	0.378	0.086	0.015	0.141	0.174	0.140	0.284	0.247
300	569	8235	0.046	0.044	0.298	0.135	0.384	0.093	0.016	0.139	0.174	0.117	0.289	0.266

*Marginal cost of public funds* Next, we test a variant of the modeling framework in which a marginal cost of public funds (MCF) is defined to penalize deviations from the deregulated scenario. In this case, the MCF is applied to both handed-out subsidies and collected taxes and is proportional to the level of public intervention. The value is arbitrarily set to 0.1. Table 7 presents the aggregate results of the new scenario. A pairwise comparison of the results of Tables 5 and 7 shows that the consequence of including the MCF is a lower intervention for ${\textit{SCC}} \le 200$ €/ton. As a result, the airline can set higher fares while preserving its competitive advantage over the slower alternatives.

**Table 7:** Approximate equilibrium solutions for an objective function that maximizes the social welfare function with values of the SCC between 100 and 300 €/ton and marginal cost of public funds equal to 0.1

SCC	$\varepsilon$	$t{\mathrm {CO}}_{2}$	Air prices		HSR prices		Regulation		Objective function			
			$r_2$	$r_3$	$r_4$	$r_5$	$t_{{\textit{TRAIN}}}$	$t_{{\textit{AIR}}}$	${\textit{SWF}}_{U}$	${\textit{SWF}}_{\pi }$	${\textit{SWF}}_{E}$	${\textit{SWF}}_{B}$
100	0.018	150.05	128.82	124.27	93.80	80.95	−0.03	0.00	−19,446	102,865	−15,005	−14
150	0.015	143.84	103.09	108.98	79.68	80.82	−15.08	0.00	−2164	89,625	−21,576	−8371
200	0.012	132.69	97.12	99.48	84.90	83.71	−22.34	14.35	−2204	87,548	−26,537	−8011
250	0.011	123.74	79.91	89.67	87.05	85.74	−30.00	30.00	−6015	82,956	−30,934	−8930
300	0.011	124.17	79.02	80.25	85.75	79.55	−30.00	30.00	−9400	79,956	−37,252	−8831

*Disaggregate policy scenario* We perform a final set of experiments where the assumption of equal policy across the population is relaxed. Specifically, the goal is to determine an optimal policy where the decision of the regulator is different between the two identified income segments. The results are presented in Table 8. We observe that both air and rail alternatives are taxed for the high-income segment, while subsidies are given to low-income rail and air travelers when ${\textit{SCC}} \le 200$ €/ton. This is explained by the higher value assigned to monetary savings by the low income segment, which is captured in the ${\textit{SWF}}_{U}$ component of the objective function. Indeed, all monetary transfers from suppliers or from the public budget to the consumers whose price sensitivity is higher (in absolute terms) than the value of the marginal utility of income result in an increase in the SWF. Only for ${\textit{SCC}} \ge 250$ €/ton does the emissions component ${\textit{SWF}}_{E}$ play a dominant role in the objective function.

**Table 8:** Approximate equilibrium solutions for an objective function that maximizes the social welfare function with values of the SCC between 100 and 300, marginal cost of public funds equal to 0.1, and policy differentiation

SCC	$\varepsilon$	$t{\mathrm {CO}}_{2}$	Air prices		HSR prices		Regulation				Objective function			
			$r_2$	$r_3$	$r_4$	$r_5$	$t_{{\textit{TRAIN}}}^H$	$t_{{\textit{TRAIN}}}^L$	$t_{{\textit{AIR}}}^H$	$t_{{\textit{AIR}}}^L$	${\textit{SWF}}_{U}$	${\textit{SWF}}_{\pi }$	${\textit{SWF}}_{E}$	${\textit{SWF}}_{B}$
100	0.019	151.37	113.83	116.45	81.16	81.16	30.00	−29.96	30.00	−8.93	0	95,298	−15,137	−577
150	0.010	143.51	97.60	106.60	97.76	84.82	29.80	−29.93	30.00	−2.18	13,355	90,521	−21,527	−9900
200	0.012	141.63	95.88	103.98	87.11	84.47	28.42	−30.00	30.00	−0.28	14,292	89,260	−28,326	−9996
250	0.011	131.18	93.36	94.79	87.52	79.52	7.88	−30.00	30.00	12.12	14,830	85,876	−32,795	−9394
300	0.014	120.05	84.72	107.15	88.24	82.70	3.33	−30.00	30.00	28.95	9898	85,444	−36,016	−7344

We remark that the presented segmentation is speculative and other socioeconomic characteristics (e.g. geographic location, age) or policy strategies (e.g. only taxation, only subsidization, revenue recycling) could be used to regulate a market at the disaggregate level.

## Conclusion

In this paper, we introduced a framework to find optimal price-based policies to regulate markets characterized by oligopolistic competition and in which consumers make a discrete choice among a finite set of alternatives. The optimization problem of the regulator is written as a mixed integer linear optimization problem, and the objective function is a social welfare function which includes the utilities of the individuals, the profits of the suppliers, the public budget and the effect of externalities. The framework accommodates general disaggregate models of demand by relying on simulation to linearize the expression of the consumers’ choice probabilities. Using a disaggregate representation of demand allows to account for product differentiation and consumer behavioral heterogeneity. However, this comes at the expense of equilibrium existence condition. To find approximate equilibrium solutions, we proposed a model-based heuristic approach based on the fixed-point iteration algorithm. Our methodology was tested on a transportation case study about an intercity travel market, where we evaluated price-based instruments in the context of emissions reduction.

The proposed framework is very general and requires limited assumptions on the specification of the used discrete choice models. This means that it can accommodate a large variety of choice models available in the literature. In particular, the use of disaggregate demand models allows to design disaggregate policies that leverage on subsidization or taxation to obtain desirable outcomes from economic, social and environmental points of view.

The following research directions could be further investigated.

Other experiments that could be performed through our framework could investigate price differentiation strategies at the supply level (Bortolomiol et al. 2021). Additionally, decisions other than price could be included in the framework, both for the suppliers and for the regulator. Examples are product assortment, capacity levels and quality changes, among others. Adapting the mathematical models is straightforward, if these variables appear as linear or integer variables in the utility functions. However, these extensions would come with additional computational complexity caused by the expanded solution space. Consequently, the applicability of our framework to large-scale problem depends on the capability to efficiently exploit the problem structure and find tight bounds or ad-hoc algorithms to such hard combinatorial problem.

A fundamental issue in public policy is the aggregation of individual utilities into a social welfare function. Different agents have different utilities and objectives that conjugate individual and social welfare. Therefore, multi-objective social welfare optimization problems cannot prescind from value judgements. In this context, our framework could be adapted to incorporate distributional preferences, policy acceptability and perceived fairness in the social welfare function.
